# Improved Deep Support Vector Data Description Model Using Feature Patching for Industrial Anomaly Detection

**DOI:** 10.3390/s25010067

**Published:** 2024-12-26

**Authors:** Wei Huang, Yongjie Li, Zhaonan Xu, Xinwei Yao, Rongchun Wan

**Affiliations:** 1 College of Computer Science, Zhejiang University of Technology, Hangzhou 310023, China; huangwei@zjut.edu.cn (W.H.); ajie001ly@gmail.com (Y.L.); znxu0315@gmail.com (Z.X.); 2 Zhejiang HOUDAR Intelligent Technology Co., Ltd., Hangzhou 310023, China; wan.rongchun@houdar.com

**Keywords:** anomaly detection, pre-trained network, deep SVDD, feature patching, unsupervised learning

## Abstract

In industrial contexts, anomaly detection is crucial for ensuring quality control and maintaining operational efficiency in manufacturing processes. Leveraging high-level features extracted from ImageNet-trained networks and the robust capabilities of the Deep Support Vector Data Description (SVDD) model for anomaly detection, this paper proposes an improved Deep SVDD model, termed Feature-Patching SVDD (FPSVDD), designed for unsupervised anomaly detection in industrial applications. This model integrates a feature-patching technique with the Deep SVDD framework. Features are extracted from a pre-trained backbone network on ImageNet, and each extracted feature is split into multiple small patches of appropriate size. This approach effectively captures both macro-structural information and fine-grained local information from the extracted features, enhancing the model’s sensitivity to anomalies. The feature patches are then aggregated and concatenated for further training with the Deep SVDD model. Experimental results on both the MvTec AD and CIFAR-10 datasets demonstrate that our model outperforms current mainstream approaches and provides significant improvements in anomaly detection performance, which is vital for industrial quality assurance and defect detection in real-time manufacturing scenarios.

## 1. Introduction

Anomaly detection, also referred to as outlier detection, involves identifying patterns in data that deviate from the expected behavior [1]. These irregular patterns are termed anomalies, outliers, inconsistencies, exceptions, aberrations, surprises, features, or contaminants, depending on the specific domain of the application. Anomaly detection has found extensive application across various fields, including fraud detection in credit card transactions, insurance, healthcare, intrusion detection in cybersecurity [2], and fault detection in safety-critical systems [3].

It is well known that deep learning technologies are effective in extracting useful features and revealing complicated input–output relationships for subsequent tasks such as classification and regression. Anomaly detection based on deep learning technologies has been widely applied in various applications due to the notable efficacy and remarkable accuracy of deep learning. Generally speaking, methods for anomaly detection in the framework of deep learning can be categorized into reconstruction-based, deep feature embedding-based, generative-based, and one-class classification-based methods. In the following, first, we will briefly review the first three categories of anomaly detection methods. Then, we will review the relevant works on one-class classification-based methods since the work in this paper belongs to the category of one-class classification methods.

As the most basic method, the objective of reconstruction-based methods is to reconstruct normal data through an encoder–decoder structure. When anomalous data are fed into a trained model, the model can still reconstruct the anomalous data as normal, i.e., the trained model cannot effectively reconstruct the anomalous input data. Reconstruction-based methods include CNN-AE [4,5], LSTM-AE [6], Conv-LSTM-AE [7], and SS-VGAE [8]. However, it was pointed out in [9] that reconstruction-based methods do not incorporate feature-level discriminative information for anomaly detection, for which inaccurate reconstruction results are sometimes yielded. To overcome this limitation, deep feature embedding-based methods have been proposed in recent years. The knowledge distillation method [10] is a typical example of the deep feature embedding-based category, which relies on transferring knowledge from a pre-trained teacher network to a smaller student network. The student network learns to mimic the teacher’s behavior on normal data. Anomalous data, which deviate from a normal data distribution, lead to larger discrepancies between the teacher and student networks, thus facilitating anomaly detection. Another approach, called Semantic Pyramid Anomaly Detection (SPADE) [11], utilizes general feature extractors based on ImageNet to extract features and then computes the similarity of these features with those of the testing data to ascertain whether a testing data point is anomalous. However, since this method relies solely on existing data for inference, the computational complexity grows with the scale of the dataset. This poses challenges for industrial deployment and applications with high real-time requirements. In addition to reconstruction-based and deep feature embedding-based methods, generative methods are primarily represented by GANs and VAEs [12], with GANs being especially prominent. GANs involve an adversarial process between two networks: the generator and the discriminator. The generator receives random samples in the latent space as input data and generates data that should be as close to the normal samples in the training set as possible. The discriminator tries to differentiate the output of the generator network from normal data as much as possible [13]. Examples of GANs include AnoGAN [13], Cycle GAN [14], and GANomaly [15].

One-class classification-based methods have shown exceptional performance in anomaly detection [16]. One-Class Support Vector Machines (OC-SVM) [17] and Support Vector Data Description (SVDD) [18] are two classical one-class classification models. These models employ a kernel function to identify an optimal boundary-separating hyperplane or boundary hypersphere in the kernel space, which is used to determine whether a testing sample is normal based on the distance to the boundary [19,20,21]. Leveraging the powerful learning capabilities of deep learning, [22] introduced the Deep SVDD (DSVDD) model for anomaly detection by integrating a deep neural network into SVDD, which effectively learns complex data patterns and achieves remarkable performance in anomaly detection. Although SVDD (or OC-SVM) and DSVDD models represent data features through kernel functions [23] and neural networks, respectively, both approaches have notable limitations. SVDD (or OC-SVM) is restricted by the choice of kernel function, while DSVDD relies on large data volumes. Thus, [24] combined a GAN and OC-SVM by training on defect-free images, extracting features from the second-to-last layer of the GAN generator, and inputting these features into OC-SVM to train a classifier. Ref. [25] proposed the Fully Convolutional Data Description (FCDD) model, which learns to map normal samples to a concentrated region in the feature space while mapping anomalous samples to locations distant from the concentrated region. In addition, Ref. [26] integrated DSVDD and AE, adding a memory module after the encoder to amplify the differences in reconstruction errors between normal and anomalous data. Ref. [27] built upon AE and Deep SVDD by treating the hypersphere center as a trainable parameter and introducing an effective anomaly score computation method. This approach enables the model to achieve outstanding performance across various anomaly detection tasks. Ref. [28] introduced BiGAN to address the scarcity of anomalous data and added penalty terms and data selection to impose radius constraints on DSVDD. This approach results in a more compact hypersphere, further improving detection performance. Ref. [29] proposed a method that jointly optimizes the VAE and SVDD models to enhance the accuracy of anomaly detection. Theoretical analysis proved its capability to prevent the “hypersphere collapse” issue. Ref. [30] took a novel approach by first defining the confidence of the DSVDD model and developing a modified power T-scaling strategy to smooth the anomaly scores of the DSVDD model. This approach enhances calibration performance without altering the original detection results. Experimental results validated the effectiveness of the proposed calibration strategy.

Although the DSVDD model has achieved promising performance in anomaly detection on both synthetic and real-world datasets, the feature learning capability of the DSVDD model is relatively weak. This is mainly because the DSVDD model primarily detects anomalies by constraining the deep features from the neural network to be embedded within a hypersphere in latent space. As a result, subtle differences between normal and anomalous data cannot be easily and precisely captured. Therefore, in this paper, we propose a novel method for anomaly detection, called Feature-Patching SVDD (FPSVDD), to capture the detailed discriminative information of anomalous data to further enhance the performance of anomaly detection. We employ a generic pre-trained feature extractor and partition the extracted features into multiple patches to enhance the perception ability of local information. By applying feature patching, the proposed model focuses on the local regions of the extracted features of the input image data, thus capturing fine-grained local anomalies more effectively. Treating each patch of extracted features as an independent entity enhances the model’s sensitivity to anomalies while reducing the impact of background noise on the overall judgment. The generic feature extractor is trained based on the ImageNet dataset. The partitions are then concatenated, and the resulting feature vectors are mapped inside a hypersphere using the DSVDD model. During the training of the DSVDD model, the optimal radius and center of the hypersphere in latent space are obtained. In the testing stage, the features of the testing data are first extracted by the pre-trained network, followed by feature patching and aggregation. Then, we can classify incoming test data as normal or anomalous based on the distance from the patched feature vector to the center of the hypersphere.

The main contributions of this paper are as follows:

- We propose a novel DSVDD model with feature patching, termed FPSVDD. Compared to the traditional DSVDD model, our feature-patching approach significantly enhances the model’s ability to capture fine-grained local information, resulting in more precise feature extraction and improved anomaly detection performance.
- We conduct extensive experiments on both the MvTec and CIFAR10 datasets. The experimental results demonstrate that the proposed FPSVDD model achieves superior anomaly detection performance and lower error rates compared to other models.
- In industrial inspection tasks, our model offers a fast inference speed, which means that it can effectively strike a balance between inference speed and detection accuracy. In other words, our model maintains high efficiency for anomaly detection without being slowed down by the increasing size of the dataset.

## 2. Method Description

**Figure 1:**
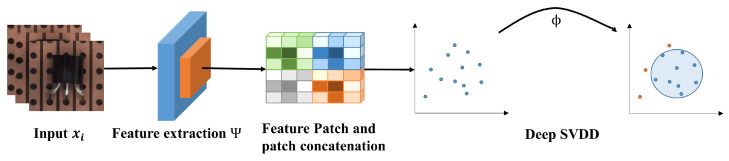
The whole structure of the FPSVDD model.

The whole structure of the proposed FPSVDD model is demonstrated in Figure 1. The input image $x_{i}$ is first processed by a generic pre-trained feature extractor to obtain the latent feature representations. Then, the latent feature representations are patched, i.e., each latent feature representation is segmented into multiple small patches. Each patched feature is then aggregated through an operation (such as average pooling) and concatenated along the channel dimension to form the patched feature vector. Finally, all patched feature vectors are fed into the DSVDD model for training to generate the smallest possible hypersphere in latent space. A patched feature vector is considered normal if it falls within the hypersphere. Otherwise, the patched feature vector is regarded as anomalous. In the testing stage, an incoming image is considered normal only if all of its patched feature vectors are detected as normal.

**Figure 2:**
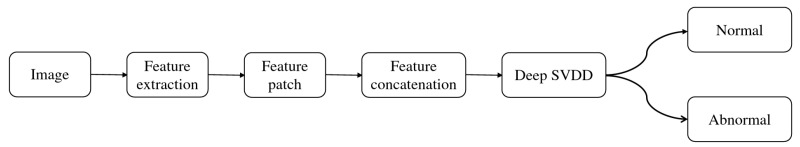
The workflow of the FPSVDD model.

Figure 2 shows the workflow of the whole model. The image is first processed through a feature extraction network to obtain the feature maps. These feature maps are then processed by the feature-patching module, which divides each feature map into smaller patches. All patches are concatenated along the channel dimension and then subsequently fed into the Deep SVDD module. Finally, the anomaly score of each patch is calculated to determine whether the input data are abnormal.

In the following, we describe each module of the FPSVDD module.

### 2.1. Feature Extraction Using Pre-Trained Network

Convolutional Neural Networks (CNNs) have proven to be an excellent choice for feature extraction from image data. In many existing studies, using normal images as the training input to the network has been regarded as the universal approach since the amount of anomalous data is usually much smaller than that of normal data in practical applications. However, due to the varying sizes of datasets encountered in industrial settings, effective feature learning can be challenging due to the risk of overfitting. Pre-trained models have been shown to be capable of learning rich visual features and higher-level semantic information [16], significantly reducing the reliance on labeled data. This is especially advantageous in anomaly detection, as pre-trained models help alleviate the challenge of acquiring anomalous samples. Consequently, employing a pre-trained network on a large dataset facilitates better generalization to the specific task, even when limited data are available for training.

In our study, we use several types of ResNet-series networks, such as ResNet50, WideResNet50, and WideResNet101, as the pre-trained models [31] to extract features. These ResNet-series networks contain an initial convolutional layer, four levels of residual blocks, and a fully connected layer. In these networks, the shallow layers capture low-level features, such as edges, lines, and textures, in the image data. The middle layers extract more complex features, such as shapes and local patterns. The higher layers capture advanced semantic features, including the overall contour of objects and class-specific information. The final layer, i.e., the fully connected layer, summarizes and compresses the high-level semantic features extracted from the entire network, providing robust classification and decision-making capabilities.

In our study, we denote *H* and *W* as the height and width of the input image. We select the extracted features from one or more levels of the residual blocks in the pre-trained network. For any image $x_{i}\in R^{H\times W\times 3}$ in the dataset, we use $\Psi_{j}\left(x_{i}\right)$ to denote the extracted features of image $x_{i}$ from the *j*-th level of the residual blocks in the pre-trained network Ψ.

### 2.2. Feature Patching and Patch Concatenation

Utilizing a general feature extractor trained on large-scale datasets, such as ImageNet [32], has proven to offer exceptional generalization capabilities [33], a process usually termed pre-training. However, the features extracted through the pre-training process often exhibit bias toward the data in the ImageNet dataset, rather than capturing the specific information needed for our task. To address this issue, we apply a patching technique to the features extracted from one or more levels of the residual blocks in the pre-trained network. By applying the patching technique to the resulting features, each patch represents a distinct segment of the feature set. Using these patches for anomaly detection mitigates the loss of spatial information and enhances the robustness of image processing.

**Figure 3:**
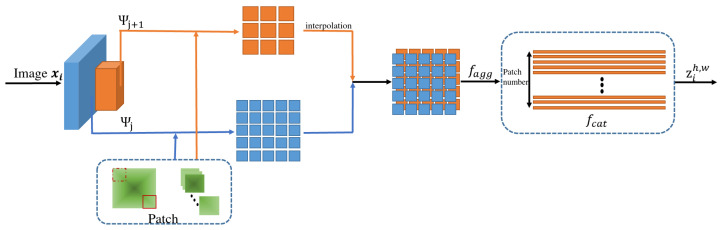
Illustration of feature patching, aggregation, and concatenation.

Figure 3 shows the whole process of feature patching, aggregation, and concatenation in the proposed FPSVDD model. Specifically speaking, we use $\Psi_{i,j}\left(h,w\right)$ to represent the feature patch of the extracted feature $\Psi_{j}\left(x_{i}\right)$ at position $h\in \{1,\ldots ,H_{j}\}$ and $w\in \{1,\ldots ,W_{j}\}$. By extracting local features and integrating global information, the receptive field of the anomaly detection model is effectively expanded. This approach enhances the robustness of deep feature processing, particularly in handling local variations within images, and improves the model’s ability to adapt to small spatial deviations.

To increase the size of the receptive field, we consider local neighbor aggregation for each patch-level feature representation. We define the neighborhood of $\Psi_{i,j}\left(h,w\right)$ as

(1) $$\begin{array}{c} N_{p}^{\left(h,w\right)}=\{\left(m,n\right)|m\in [h-\left\lfloor \frac{p}{2}\right\rfloor ,\ldots ,h+\left\lfloor \frac{p}{2}\right\rfloor ],n\in \left[w-\left\lfloor \frac{p}{2}\right\rfloor ,\ldots ,w+\left\lfloor \frac{p}{2}\right\rfloor ]\right\}, \end{array}$$

where *p* denotes the patch size.

Then, the neighborhood-aware features $u_{i,j}^{h,w}$ at position (h,w) are obtained by aggregating the features in the neighborhood $N_{p}^{\left(h,w\right)}$ using an aggregation function $f_{agg}$, which can be expressed as

(2) $$\begin{array}{c} u_{i,j}^{h,w}=f_{agg}\left(\left\{\Psi_{i,j}\left(h^{\prime },w^{\prime }\right)|\left(h^{\prime },w^{\prime }\right)\in N_{p}^{\left(h,w\right)}\right\}\right). \end{array}$$

In our model, we utilize adaptive average pooling to implement the aggregation function $f_{agg}$ for each patch.

It should be mentioned that features can be extracted from multiple levels of the residual blocks in the pre-trained network. The number of patches in both the height and width directions (i.e., $H_{j}$ and $W_{j}$) from the different levels of the residual blocks are distinct from each other. Therefore, we cannot directly fuse the features from the different levels of the residual blocks. To effectively fuse $u_{i,j}^{h,w}$ from the multiple levels of the residual blocks, a linear interpolation technique is applied to all neighborhood-aware features, except those with the largest size. Then, after linear interpolation, the neighborhood-aware features from all levels of the residual blocks in the pre-trained network have the same size.

Finally, by concatenating the adjusted and unadjusted neighborhood-aware features $u_{i,j}^{h,w}$ along the channel dimension and from the different levels of the residual blocks, we obtain the final feature representation vector $z_{i}^{h,w}$:

(3) $$\begin{array}{c} z_{i}^{h,w}=f_{cat}\left(u_{i,j}^{h,w}\right). \end{array}$$

### 2.3. Deep SVDD (DSVDD) Model

After concatenating the patched features from one or more levels of the residual blocks in the pre-trained network, we use the DSVDD model to distinguish anomalous patch data from normal patch data. In the DSVDD model, the feature representation $z_{i}^{h,w}$ is mapped into latent space H via a deep neural network $\Phi (z_{i}^{h,w};W)$. Here, $W=\{W^{1},W^{2},\ldots ,W^{L}\}$ denotes the sets of weights in the network and *L* is the number of network layers. In the latent space H, DSVDD models the distribution of normal patched features using a center point c (i.e., the center of the hypersphere) and a radius *R*. The objective of the DSVDD model is to construct a hypersphere with the smallest possible radius such that normal patched features are as close as possible to the center and lie within the hypersphere, while anomalous patched features lie outside the constructed hypersphere. In the training stage, DSVDD seeks to minimize the following objective function:

(4) $$\begin{array}{c} \min_{R,W}R^{2}+\frac{1}{vNH^{o}W^{o}}\sum_{i=1}^{N}\sum_{h=1}^{H^{o}}\sum_{w=1}^{W^{o}}\max\{0,∥\phi \left(z_{i}^{h,w};W\right){-c∥}^{2}\}+\frac{\lambda }{2}\sum_{l=1}^{L}{∥W^{l}∥}_{F}^{2}. \end{array}$$

In (4), $\phi (z_{i}^{h,w};W)$ represents the feature vector in the latent space H. $H^{o}$ and $W^{o}$ are the number of patches after linear interpolation in the height and width directions, respectively. *R* and c denote the radius of the constructed hypersphere and the center point of the hypersphere in latent space, respectively. *v* and λ are two positive coefficients. *N* is the number of data points in the training set. Finally, ${∥•∥}_{F}$ denotes the Frobenius norm.

The first term of (4) aims to minimize the radius of the hypersphere that contains normal feature vectors in the latent space H. In addition, the second term seeks to minimize the distance of feature vectors to the center c. The last term is the weight decay regularize, which mitigates overfitting of the neural network by constraining its complexity.

### 2.4. Training and Inference

In the training stage, the normal data in the training set are patched, aggregated, and concatenated, as described in Section 2.2, to obtain the patched feature representation vector $z_{i}^{h,w}$. The representation vectors are then input into the DSVDD model, where the neural network maps $z_{i}^{h,w}$ into the latent feature vector inside a hypersphere centered at c. The network $\phi (z_{i}^{h,w};W)$ continuously adjusts its weights to minimize the distance between normal patched data points and the center c in the latent space, thus ensuring that the distribution of normal patched data is as close as possible to the center of the hypersphere.

At the beginning of the training stage, the center point c is initialized as the mean of the initial feature vectors mapped from $z_{i}^{h,w}$, under the assumption of no interference from anomalous data. As training progresses, normal patch data increasingly concentrate within the hypersphere in the latent space, while anomalous patch data are more likely to fall outside the hypersphere.

In the inference stage, a given testing image $x_{test}$ is processed through the feature extractor Ψ, feature aggregator $f_{agg}$, and feature concatenator $f_{cat}$ to obtain the final patched feature representation vectors $\left\{z_{test}^{h,w}\right\}$. The anomaly score of each $z_{test}^{h,w}$ can then be defined based on the distance from the patched feature vector $\phi (z_{i}^{h,w};W)$ to the center of the hypersphere in the latent space H:

(5) $$\begin{array}{c} s\left(z_{test}^{h,w}\right)={∥\phi \left(z_{test}^{h,w};W^{*}\right)-c∥}^{2}. \end{array}$$

In (5), $W^{*}$ denotes the network weights after the training process. The DSVDD model establishes the optimal center and radius of the hypersphere by learning the distribution of the normal patched feature vectors. When each feature of a testing image $x_{test}$ is split into multiple patches, the distances between all patched feature vectors and the hypersphere center in the latent space are calculated to determine whether each patched feature vector falls within the hypersphere. If a patched feature vector is inside the hypersphere, this feature vector is classified as normal. Otherwise, this feature vector is classified as anomalous. A testing image $x_{test}$ is considered normal only when all the patched feature vectors $\left\{z_{test}^{h,w}\right\}$ generated from $x_{test}$ have anomaly scores smaller than the radius of the hypersphere in the latent space. Otherwise, if at least one patched feature vector is found to lie outside the hypersphere, the corresponding testing image is considered anomalous.

## 3. Experiments

In this section, we evaluate the performance of the FPSVDD model on the task of anomaly detection. To verify the applicability and efficacy of the FPSVDD model, we conducted several experiments on the MvTec and CIFAR10 datasets, comparing the performance of the FPSVDD model with that of several other baseline models and state-of-the-art techniques. The Adam optimizer was used in the FPSVDD model with a learning rate of $10^{-4}$. For all datasets, the batch size was set to 8, and the weight decay hyperparameter was set to $\lambda =10^{-6}$.

### 3.1. Dataset Description

Here, we provide brief introductions to the MvTec and CIFAR10 datasets.

We first carried out experiments on the MvTec anomaly detection dataset [34], which is a well-established benchmark dataset for unsupervised anomaly detection in industrial settings. Released by MVTec Software GmbH, this dataset was specifically designed to evaluate the performance of anomaly detection methods in industrial inspection tasks. The MvTec dataset comprises 15 categories of industrial products, each containing both normal and anomalous samples. Defects in anomalous samples include surface scratches, deformations, cracks, stains, and material losses. These defects are commonly encountered in industrial production, making the dataset highly relevant for practical applications. In our experiments, we split the MvTec dataset into a training set and a testing set. The training set comprised only normal samples, allowing the model to learn the typical characteristics of normal objects. The testing set included both normal and anomalous samples, accompanied by pixel-level annotations for the anomalies, enabling a precise evaluation of detection performance.

The second dataset we utilized was the CIFAR10 dataset [35], which includes 10 different kinds of objects (i.e., 10 classes) used in daily life. This dataset consists of 60,000 32×32 RGB color images. In each experiment on this dataset, we designated one class as normal and the others as anomalous. The training and testing sets consisted of 50,000 and 10,000 images, respectively. The training set only contained classes designated as normal, while the testing set contained all 10 classes in the dataset.

### 3.2. Evaluation Metrics

To effectively evaluate the performance of the FPSVDD model, we employed the Area Under the Receiver Operating Characteristic Curve (AUROC) as the metric to assess the performance of all anomaly detection models in our study. The AUROC is a commonly used metric as an indicator of discrimination accuracy for evaluating classification models, especially in binary classification tasks. The ROC curve illustrates the relationship between the true positive rate (TPR) and the false positive rate (FPR) across various threshold settings, with the AUROC representing the area under this curve.

### 3.3. Experimental Settings

In the experiments, we first tested three different kinds of backbone networks as the pre-training network to extract features, including WideResNet50, WideResNet101, and ResNet50. Through these experiments, it was found that WideResNet50 was the optimal backbone network to extract features. Therefore, we chose WideResNet50 as the backbone network for pre-training to be included in our proposed FPSVDD model. Then, since WideResNet50 contains multiple levels of bottleneck residual blocks, we also investigated which combination of levels was most beneficial for enhancing anomaly detection performance. After determining the optimal combination of levels to extract features, we then compared the performance of the FPSVDD model with that of other state-of-the-art models.

The MvTec dataset consists of three-channel RGB and grayscale images with resolutions ranging from 700×700 to 1000×1000 pixels, while the CIFAR10 dataset contains three-channel RGB images with a resolution of 32×32 pixels. During the initial preprocessing of both the MvTec and CIFAR10 datasets, we first resized each image to 329×329 pixels, followed by a center crop to 288×288 pixels. We use Conv2(*k*,*s*,*c*) to denote a 2D convolutional layer, where *k*, *s*, and *c* represent the kernel size, stride, and number of channels, respectively. We extracted features from one or more levels of the bottleneck residual blocks in WideResNet50. Each level consisted of multiple bottleneck residual blocks. In each level, the first bottleneck residual block was structured as Conv2(1,2,1024)-BN-Conv2(3,1,1024)-BN-Conv2(1,1,1024)-BN-ReLU-downsample(Conv2(1,2,1024)-BN) with a skip connection, while the remaining bottleneck residual blocks had a similar structure to the first block but without the downsampling operation at the end. Downsampling in the first bottleneck residual block of each level was used to reduce the spatial dimensions of the feature map while preserving the information of the extracted multi-level features. After passing through the pre-trained feature extractor, feature patching was applied to the extracted features, followed by channel-wise aggregation for these feature patches. The patch size was fixed at 3×3 for comparison in our experiments. Finally, we concatenated the aggregated feature patches and reduced the final dimensionality to 1536.

In the subsequent DSVDD module, we used a fully connected layer for dimensionality reduction, which consisted of a linear layer followed by batch normalization (BN), a Leaky ReLU activation function, and another linear layer. The initial weights of the linear layers were all set to $0.5\times 10^{-6}$. After passing through the fully connected layer, the dimensionality of the data was reduced to 32, and these data were then used to train the DSVDD model to obtain the center and radius of the hypersphere in the latent space H. The center of the hypersphere was initialized as the mean of the feature vectors in the latent space at the beginning of the training epochs.

In our experiments, we compared the proposed FPSVDD model with several other deep learning-based anomaly detection models, including DSVDD [22], OC-SVM [17], ITAE [36], GANomaly [15], SPADE [11], CAVGA-Rw [37], and IAE-LSTM-KL [38], which are summarized as follows:

- The core idea of OC-SVM (One-Class Support Vector Machine) is to construct a separating hyperplane that separates the majority of normal samples from anomalous ones in the kernel space. Specifically, OC-SVM seeks to find a hyperplane that encompasses as many normal data points as possible on one side, while the data points on the other side are regarded as anomalies.
- ITAE (Inverse-Transform Autoencoder) employs a traditional autoencoder (AE) structure, which enhances the model’s ability to express complicated data structures by processing the output of the encoder through some form of inverse-transformation or mapping method. This ensures that certain structural features of data are either strengthened or preserved during reconstruction.
- GANomaly employs an adversarial autoencoder within an encoder–decoder–encoder structure to model the distribution of training data within both the image level and the latent vector space.
- SPADE (Semantic Pyramid Anomaly Detection) first utilizes a pre-trained deep neural network, such as ResNet trained on ImageNet, to extract image features. Then, the KNN (*K*-Nearest Neighbors) method is employed to find the *K*-nearest normal images for a given target image. Finally, correspondences between normal images and the target image are found based on a multi-resolution feature pyramid to detect anomalies.
- CAVGA-Rw (Conditional Adversarial Variational Generative Adversarial Network with Regularization) adopts a weakly supervised approach. This model involves VAE training on one side and introduces a binary classifier (normal/anomalous) on the other side, utilizing binary cross-entropy loss to train the classifier. Based on the prediction results of the binary classifier, the CAVGA-Rw model minimizes the attention corresponding to the anomalous class while enhancing the attention for the normal class.
- The IAE-LSTM-KL model enhances the feature representation of normal data by capturing long-term dependencies through an LSTM module. The model also applies KL divergence to penalize the feature inputs to the SVDD module. Experimental results show that the IAE-LSTM-KL model yields remarkable detection accuracy for anomalies.

### 3.4. Experimental Results and Analysis

As discussed above, the FPSVDD model contains the pre-training backbone network, which includes multiple levels of residual blocks. The choice of the proper type of backbone network should be determined first in order to extract useful features for further processing by the DSVDD model. On the other hand, the features extracted from different levels of the bottleneck residual blocks have different characteristics. For example, shallow levels often capture low-level features such as edges and textures, while deeper levels extract high-level features such as object shapes and structures. Therefore, it is of great importance to extract features from the most suitable level(s) of the residual blocks.

First, we conducted experiments under various backbone networks for pre-training to extract useful features for further processing on the MvTec dataset. The backbone networks we employed included ResNet50, WideResNet50, and WideResNet101. These backbone networks differ in their feature extraction capabilities, generalization abilities, and adaptability to various types of data. As shown in Table 1, WideResNet50 yielded the highest AUROC values. ResNet50 is a classical residual network known for its simple structure and broad adaptability, making it a suitable choice for pre-training in various visual-related tasks. In comparison, WideResNet50 is able to further enhance feature extraction capabilities by increasing the number of channels. The wider convolutional layers of WideResNet50 capture more detailed information, which is helpful for extracting richer feature representations. On the other hand, WideResNet101, with a deeper network structure and wider convolutional layers than WideResNet50, offers even stronger feature learning capabilities. However, the increased number of parameters in WideResNet101 may cause the model to capture noise and irrelevant information from training data, potentially leading to overfitting and performance deterioration of the model.

We also validated the performance of two other pre-trained networks. The first was the fasterrcnn_resnet50_fpn_coco model pre-trained on the COCO dataset, and the second was the efficient_b7 model pre-trained on the JFT-300M and ImageNet-1k datasets. The experimental results based on the two pre-trained networks are presented in Table 1. The results show that ResNet50 yielded a higher AUROC value than networks using both the fasterrcnn_resnet50_fpn_coco and efficient_b7 models.

Then, on the pre-trained WideResNet50 network, features were extracted from one or more levels of the bottleneck residual blocks. We indexed the first three levels of the bottleneck residual blocks in WideResNet50 as 1, 2, and 3, respectively, and present the AUROC values in Table 2, which represent the performance when features were extracted from different combinations of levels of the residual blocks. It can be observed that extracting features only from level 3 yielded the highest AUROC value. Adding additional features (for example, using level 2 + level 3) was not beneficial for further enhancing anomaly detection performance. Therefore, in the subsequent experiments comparing the performance of the FPSVDD model with that of other competing models, we adopted WideResNet50 as the backbone network in the FPSVDD model to extract features from level 3 of the residual blocks in WideResNet50.

Table 3 and Table 4 show the AUROC values of our proposed FPSVDD and other competing models on the MvTec and CIFAR10 datasets, respectively. As shown in Table 3, FPSVDD achieved exceptional detection performance in most categories, with an overall average AUROC of 91.6%. Notably, in the Leather and Bottle categories, our model achieved detection accuracies of up to 99.7% and 100%, respectively. In addition, it should be noted that the MvTec dataset is mainly categorized into two categories: texture and object category. The texture category includes classes such as carpet, grid, and leather, while the object category includes classes such as bottle, cable, and capsule. For the texture category, the DSVDD model achieved an average AUROC of 58.8%, which was lower than that for the object category. This can be attributed to the DSVDD model’s limited ability to capture detailed image information. In contrast, since the FPSVDD model divides the image into multiple small patches, the aggregation of patch-based features effectively captures local information and enhances the model’s sensitivity to subtle anomalies. Therefore, our proposed FPSVDD model achieved a significant increase in the average AUROC for the texture category (94.24%) compared to the object category (88.9%).

In each anomaly detection experiment on the CIFAR10 dataset, we designated one class as normal, while the other nine classes were regarded as anomalous. For each class identified as normal, a corresponding model for anomaly detection was trained. The results in Table 4 indicate that feature patching significantly improved model performance, achieving excellent results in the airplane, car, and truck categories but performing slightly worse in the bird, cat, and deer categories compared to the other models. Overall, our proposed FPSVDD model achieved a higher average AUROC value on the CIFAR10 dataset, which indicates that the FPSVDD model is efficient for anomaly detection on the CIFAR10 dataset.

Next, we investigated the stability of all models under study in the training process. Stability is an important indicator for measuring the performance of anomaly detection models. A stable model is usually preferred since it is expected that an anomaly detection model is robust to adverse factors that may negatively affect anomaly detection performance. For example, the images in the training set may differ greatly, or there could be some noise or outliers in the images. If the chosen anomaly detection model is sensitive to such adverse factors, the trained model would fail to capture the intrinsic features of image data. As a result, the model’s detection performance on the testing data may be compromised.

As shown in Table 5, we also conducted experiments at different levels of noise. Gaussian noise was added to each pixel of every image in the dataset. "Low Noise" refers to Gaussian noise with a standard deviation of 10, while "High Noise" refers to Gaussian noise with a standard deviation of 50. In Table 5, we can observe that at a low level of Gaussian noise, the performance of the FPSVDD model only decreased by 1%, meaning its performance was still higher than that of all other models on the clean MvTec dataset (as illustrated in Table 3). At a low level of Gaussian noise, FPSVDD still achieved an average AUROC of 87.6%, which was lower only than that of CAVGA-Rw but higher than that of the other models on the clean MvTec dataset.

**Figure 4:**
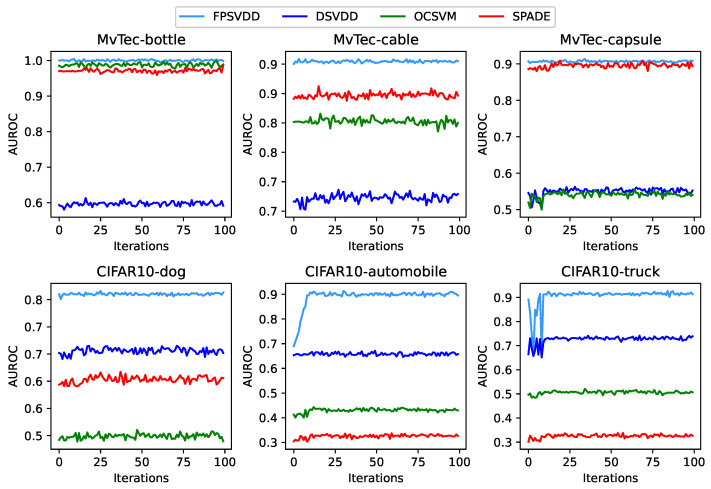
AUROC values versus training epochs on different datasets. The dataset and the normal class within each dataset are indicated above each subplot. For example, “CIFAR10-truck” denotes that the results in this subplot are based on the truck class as the normal class in the CIFAR10 dataset.

To examine the stability of our proposed FPSVDD model and several other competing models in our study, we plotted the iteration evolution of the AUROC values for all models under consideration on both the MvTec and CIFAR10 datasets, as demonstrated in Figure 4. It can be observed that, although there were some fluctuations in the early stages of training, the overall AUROC values of the FPSVDD model were consistently higher than those of the other models throughout the training process. This result indicates that the FPSVDD model presents better stability than other competing models.

Then, to gain insights into the efficiency of models in practical applications, we also compared the inference times for some anomaly detection methods on the MvTec dataset, as illustrated in Table 6. The inference time of the DSVDD model (pre-trained on WideResNet50) was relatively low since the network weights and the radius of the hyperparameters were fixed after the training stage. Therefore, during the inference stage, we only needed to calculate the distance between the features mapped from the testing image patch and the center of the hypersphere in the latent space, allowing for a quick determination of the anomaly score. In contrast, SPADE (pre-trained on WideResNet50) required extensive computation time to calculate the distances between the testing image and the existing images in the training set. While this is acceptable for smaller datasets, the computation time increases with the size of the datasets, which makes it unfeasible for real-time inspections in industrial applications. Although the FPSVDD model involves feature patching, aggregation, and concatenation in the testing stage, the FPSVDD model demonstrated superior inference efficiency over SPADE. Since IAE-LSTM-KL and GANomaly do not involve pre-trained modules, their inference speeds were faster. However, the two models performed significantly worse compared to our proposed FPSVDD model. Despite the more complicated processing involved, FPSVDD effectively utilizes local information, keeping the inference time within a reasonable range.

Finally, we also conducted experiments evaluating the impact of the patch size *p* on the performance of anomaly detection for the FPSVDD model. The results are illustrated in Table 7. It can be observed that medium-sized patches (i.e., p=3) yielded a higher AUROC value. This is mainly due to the discrepancy in the characteristics of the extracted features using different patch sizes. Small patches focus on capturing fine local features while overlooking the macrostructure and patterns in the image data. In contrast, choosing larger patch sizes results in the loss of fine-grained details in the image data. Medium-sized patches can strike a balance by extracting detailed features without overlooking global information, enabling the FPSVDD model to effectively adapt to both large-scale anomalies and subtle irregularities.

Overall, compared to traditional DSVDD, our approach places a greater emphasis on local details when processing data. By leveraging the powerful feature extraction capabilities of pre-trained networks, the proposed model extracts features and uses patch operations to treat local regions as individual units. This enables the model to capture subtle differences more sensitively, and any changes in local areas do not affect the feature extraction of other patches, thereby enhancing robustness. By adjusting the patch size, the model achieves a balance between local details and global context, leading to improved performance. We also compared processing speeds. In the future, it would be worthwhile to explore rapid and accurate anomaly detection when acquiring image data through sensors.

## 4. Conclusions

In this paper, we propose a novel model called Feature-Patching SVDD for unsupervised anomaly detection. This model first employs the high representational ability of features extracted from the pre-trained backbone network on ImageNet. Then, the extracted features are split into multiple small patches of appropriate sizes to enhance the model’s ability to perceive local information in the image data. After some manipulations on the patched features, the resulting patched feature vectors are fed into the deep SVDD model, which yields a compact hypersphere containing all normal patched feature vectors in the latent space. A testing image is considered normal only when all its patched feature vectors lie within the hypersphere. Experimental results show that the proposed FPSVDD model outperforms other competing methods in anomaly detection.

## Figures and Tables

**Table 1:** Performance of anomaly detection using different backbone networks for pre-training on the MvTec dataset.

Backbone	AUROC (%)
fasterrcnn_resnet50_fpn_coco	82.7
efficient_b7	84.1
ResNet50	87.6
WideResNet50	**91.6**
WideResNet101	88.4

**Table 2:** Anomaly detection performance when features are extracted from different combinations of levels of the residual blocks in WideResNet50.

Level 1	Level 2	Level 3	AUROC (%)
*√*			85.3
*√*	*√*		86.9
	*√*		87.6
	*√*	*√*	89.9
		*√*	**91.6**
*√*	*√*	*√*	88.3

**Table 3:** AUROC values (%) on the MvTec dataset.

Model	DSVDD	GANomaly	OC-SVM	ITAE	SPADE	CAVGA-Rw	IAE-LSTM-KL	Ours
Carpet	61.0	69.9	62.7	70.6	92.8	88.0	66.6	**94.3**
Grid	53.2	70.8	41.0	88.3	47.3	84.0	**90.4**	80.6
Leather	52.1	84.2	88.0	86.2	95.4	89.0	66.2	**99.7**
Tile	62.1	79.4	87.6	73.5	96.5	97.0	79.6	**98.7**
Wood	65.4	83.4	95.3	92.3	95.8	79.0	95.9	**97.9**
**Avg.Text**	58.8	77.5	74.9	82.2	85.5	87.4	79.7	**94.2**
Bottle	59.8	89.2	99.0	94.1	97.2	96.0	94.5	**100**
Cable	67.9	75.7	80.3	83.2	84.8	**92.0**	85.0	91.6
Capsule	55.4	73.2	54.4	68.1	89.7	**93.0**	71.6	91.0
Hazelhut	65.4	78.5	91.1	85.5	88.1	**97.0**	75.7	80.9
Metal Nut	65.8	70.0	61.1	66.7	71.0	82.0	76.1	**90.6**
Pill	50.9	74.3	72.9	78.6	80.1	**86.0**	75.8	75.7
Screw	71.1	74.6	74.7	**100**	66.7	81.0	86.9	68.7
Toothbrush	64.3	65.3	61.9	**100**	88.9	89.0	**100**	97.5
Transistor	58.7	79.2	56.7	84.3	90.3	**99.0**	90.0	94.0
Zipper	57.6	74.5	51.7	87.6	96.6	96.0	87.7	**99.0**
**Avg.Obj**	61.7	75.4	70.4	84.8	85.3	**91.1**	85.9	88.9
**Average**	60.7	76.2	72.6	83.5	85.5	89.2	82.8	**91.6**

**Table 4:** AUROC values (%) on the CIFAR10 dataset.

Model	DSVDD	GANomaly	OC-SVM	ITAE	SPADE	CAVGA-Rw	IAE-LSTM-KL	Ours
airplane	61.7	**93.5**	63.0	67.4	32.7	65.3	78.0	90.3
automobile	65.9	60.8	44.0	60.9	32.1	78.	68.8	**90.3**
bird	50.8	59.1	64.9	60.5	49.3	**76.1**	61.3	72.8
cat	59.1	58.2	48.7	67.1	51.8	**74.7**	64.8	74.1
deer	60.9	72.4	73.5	67.0	26.8	**77.5**	70.6	71.2
dog	65.7	62.2	50.0	65.5	60.5	55.2	65.0	**76.8**
frog	67.7	**88.6**	72.5	70.7	24.1	81.3	75.9	82.3
horse	67.3	56.0	53.3	69.3	48.8	74.5	65.8	**81.2**
Ship	75.9	76.0	64.9	69.7	30.0	70.1	79.8	**84.5**
truck	73.1	68.1	50.8	61.0	32.8	74.1	77.7	**91.6**
**Average**	64.8	69.5	58.2	65.6	38.9	73.7	70.8	**81.6**

**Table 5:** Performance of anomaly detection under different noise levels.

Noise	AUROC (%)	Descending (%)
No Noise	91.6	-
Low Noise	90.6	1
High Noise	87.6	4

**Table 6:** Comparison of inference times for anomaly detection on the MvTec dataset.

Model	SPADE	DSVDD	FPSVDD	IAE-LSTM-KL	GANomaly
Inference time (s)	6.45	0.03	0.25	0.09	0.01

**Table 7:** Performance of anomaly detection under different patch sizes.

Patch Size *p*	AUROC (%)
1	87.6
2	88.6
3	**91.6**
4	84.9
5	83.5

## Data Availability

The original contributions presented in this study are included in the article. Further inquiries can be directed to the corresponding author.
